# The polyphenol/saponin-rich *Rhus tripartita* extract has an apoptotic effect on THP-1 cells through the PI3K/AKT/mTOR signaling pathway

**DOI:** 10.1186/s12906-021-03328-9

**Published:** 2021-05-27

**Authors:** Hajer Tlili, Anca Macovei, Daniela Buonocore, Manuela Lanzafame, Hanen Najjaa, Anita Lombardi, Andrea Pagano, Maurizia Dossena, Manuela Verri, Abdelkarim Ben Arfa, Mohamed Neffati, Enrico Doria

**Affiliations:** 1 Laboratory of Pastoral Ecosystems and Valorization of Spontaneous Plants and Microorganisms, Institute of Arid Regions (IRA), Tunis, Medenine Tunisia; 2 Department of Biology and Biotechnology “L. Spallanzani”, University of Pavia, via Ferrata 9, 27100 Pavia, Italy; 3 Institute of Molecular Genetics IGM CNR, Pavia, Italy

**Keywords:** Plant extracts, Medicinal plants, *Rhus tripartita*, mTOR pathway, Anticancer properties, Apoptosis, Leukaemia

## Abstract

**Background:**

Hyperactivation of mechanistic target of rapamycin (mTOR) signaling pathway is involved in the regulation of cellular growth, proliferation, and more in general, is a common phenomenon in most types of cancers. Thus, natural substances targeting this pathway can be of great therapeutic potential in supporting the treatment of tumor patients. *Rhus tripartita* (Ucria) Grande is a plant growing in desertic areas which is traditionally used for the treatment of several diseases in Tunisia. In the present work, the biochemical profile of the main compounds present in the plant leaf extract was determined and the anti-leukemic potential of the plant extracts against acute monocytic leukaemia (AML) THP-1 cells was investigated.

**Methods:**

After HPLC identification of some phenolic compounds present in the plant extract and the quantification of saponin content, the cytotoxic effect of Rhus tripartita extracts on THP-1 cell culture was evaluated using the colorimetric MTT assay for cell viability. THP-1 cells were incubated with medium containing the relative IC_50_ concentrations of total plant extract, saponin extract and some standard compounds (rutin (R); kaempferol (K); mixture of catechin, epicatechin, and epicatechin-gallate (CEEG); ellagic acid (EA). Finally, qRT-PCR and western blotting analysis were used to evaluate the effect of some flavonoids present in a crude extract of polyphenols and the total extract of saponins on cell survival and apoptosis.

**Results:**

Analysis of expression level of some gene (*PIK3CA, PTEN, AKT1, mTOR, EIF4E, RPS6KB1*, and *TSC1*) involved in the mTOR pathway and the phosphorylation of S6 and AKT proteins allowed to observe that a total Rhus tripartita extract and some of the compounds found in the extract controls THP-1 cell proliferation and apoptosis via regulation of the PI3K-Akt-mTOR signaling pathway.

**Conclusion:**

*Rhus tripartita*-induced inhibition of cell cycle and induction of apoptosis may involve the mTOR pathway. Therefore, *Rhus tripartita* extract may be a useful candidate as a natural anti-cancer drug to support the treatment of AML.

**Supplementary Information:**

The online version contains supplementary material available at 10.1186/s12906-021-03328-9.

## Background

A large number of scientific reports have demonstrated that medicinal plants from different geographical areas of the world are a rich source of biologically active compounds that can support treatments of various diseases, including some types of cancers. Plants, in fact, have provided an endless supply of secondary metabolites or phytochemicals increasingly used against various types of cancer [1]. In particular, aromatic plants used in folk medicine and in phytotherapy contain a high number of chemically diverse and structurally complex polyphenols holding a number of benefits such as an added economical value, lack of toxic effects, and inherent biological activities that make them attractive candidates for new therapies [2]. Belonging to the polyphenolic molecules group, flavonoids include almost 10,000 different compounds organized in subclasses as flavonols, flavan-3-ols, anthocyanins, flavanones, flavones, isoflavones and proanthocyanidins. These phytochemical classes show antibacterial, antiviral, anti-inflammatory, antilipidemic, or antidiabetic activities, along with neuroprotective, hepatoprotective or cardioprotective properties [3]. Moreover, flavonoids can act as therapeutic tool for cancer chemoprevention; research on flavonoids showed major developments in anticancer drug discoveries with potential to destroy cancer cells through apoptotic induction [4, 5].

The mechanistic target of Rapamycin (mTOR, also known as mammalian target of Rapamycin) is a highly conserved protein kinase and a central controller of cell growth and metabolism [6]. The PI3K/AKT/mTOR signaling pathway regulates various physiological functions such as cell cycle progression, transcription, mRNA translation, differentiation, apoptosis, autophagy, motility, and metabolism [7]. Hyperactivation of mTOR results in an increase in cell growth and induce the entrance of some cells into the cell cycle [8]. Aberrant PI3K/AKT signaling, which is a major cause of hyperactivation of the mTOR pathway, has been reported in many human cancers, and is contributing to both cancer pathogenesis as well as therapy resistance [9]. Loss-of-function due to mutations in tumor suppressors, such as phosphatase and tensin homolog (PTEN), tuberous sclerosis 1/2 (TSC1/2), neurofibromin 1/2 (NF1/2), or oncogenic mutations in KRAS, PIK3CA, or AKT are the most common causes of mTOR signaling hyperactivity [10]. Aberrant activation of the PI3K/AKT/mTOR axis, which deregulates cellular functions such as proliferation, metabolism, and survival, has been associated with the progression and pathogenesis of a broad spectrum of various types of human cancer, including AML (acute myeloid leukaemia) [11]. AML is a heterogeneous disease resulting from multiple genetic and epigenetic abnormalities that affect differentiation, proliferation and apoptosis of myeloid cells [12]. The traditional treatment for leukaemia is chemotherapy; however, chemotherapy has serious side effects and may result in treatment failure due to treatment-associated mortality or the emergence of drug resistance [13]. A rare population of therapy-resistant cells are believed to be the origin of relapse, termed leukaemia stem cells (LSCs) or leukaemia-initiating cells (LICs). These cells acquire enhanced self-renewal capacity and exhibit a block in differentiation. Thus, eliminating LSCs may prevent relapse. Hyperactivation of PI3K/Akt/mTOR has been associated with attenuated sensitivity to chemotherapy and growing evidence suggests that targeting key components of this pathway may represent an effective treatment to kill AML LSCs [14]. Therefore, investigating novel approaches for inhibiting the PI3K/AKT/mTOR signaling pathway in order to develop targeted therapeutics while limiting chemotherapy side effects, is vital for increasing treatment efficacy and improving prognosis in patients [15]. Plant polyphenols, and flavonoids in particular, play a crucial role in inhibiting various kinases involved in PI3K/AKT/mTOR signaling cascades [1]. Due to their high contents in phenols, flavonoids and other phytochemicals, *Rhus* species are widely used in both modern and traditional medicine. In Tunisia, the genus *Rhus* is mainly represented by the species *Rhus tripartita* (Ucria) Grande; extracts of this genus of plant showed antimalarial [16], antimicrobial [17], antitumorigenic [18, 19], antioxidant [20], hypoglycemic [21] and anti-proliferative [21, 22] effects. As evidenced in this recent study [22], alcoholic extracts of a Tunisian variety of *Rhus tripartita* present high levels of polyphenols and flavonoids. According to this evidence, in the present work, the biological activity of a biochemically characterized *Rhus tripartita* extract on mTOR signaling pathway and cell survival in THP-1 leukaemia cell line is described. In particular, the effect of some flavonoids present in a crude extract of polyphenols and the total extract of saponins on cell survival and apoptosis was evaluated. Analysis of expression level of some gene (*PIK3CA, PTEN, AKT1, mTOR, EIF4E, RPS6KB1*, and *TSC1*) involved in the mTOR pathway and the phosphorylation of S6 and AKT proteins indicated how the *Rhus tripartita*-induced inhibition of cell cycle and induction of apoptosis may involve the mTOR pathway.

## Methods

### Plant material

Leaves of *Rhus tripartita* (Ucria) D.C., collected during the vegetative phase, was provided by the Institut des Regions Arides (IRA) in Medenine, Tunisia, and authenticated by botanist Dr. Mohammed Neffati according to the “Flora of Tunisia” catalogue [23]. Voucher specimen was deposited at the herbarium of the IRA (accession number: IRABS1830). The harvested plant samples were processed (powdered at around 100 mesh, using a Retsch S/S Cross Beater Hammer Mill Sk1) then dried in the shade at room temperature for two weeks and finally stored under dark condition until use. The investigations regarding the plant were carried out following the recommendations of the Convention on Biological Diversity, signed by 150 government leaders at the 1992 Rio Earth Summit.

### Preparation of total plant extract

An amount of 30 g of the finely powdered leaves was macerated with acetone 70% (150 ml) for 7 days under continuous shaking conditions (50 rpm). The extract obtained at the end of the maceration process was dried using a rotavapor (Rotavapor® R-300, Buchi Italia, Italy) and the dry residue was resuspended in ethanol 50%. The obtained extract was then used for further analyses. (HPLC and toxicity assay).

### Extraction of saponins fraction

Total saponin content (percent yield) was determined by gravimetric method as described by Kaur et al. (2015) [24]. An amount of plant material (20 g) was macerated in methanol 70% (100 ml) for 24 h in dark conditions and then partitioned in a water and n-butanol (1:1 ratio) solution. This obtained solution was poured into the separator funnel and kept for 2 h. The upper n-butanol layer was separated and the solvent was evaporated to obtain crude saponin extract.

### HPLC analysis

Polyphenol content from the plant extract (ethanol 50%) obtained as previously described, was assayed by an HPLC system (Kontron Instrument 420 system) (Kontron Instruments, Munich, Germany) equipped with a reverse phase C18 column (SepaChrom® - Robusta, 100A, 5 μ, 250 × 4.6 mm) and UV detector. The mobile phase, fluxed at a rate of 0.8 ml / min, consisted of 4% acetic acid (solvent A) and pure methanol (solvent B) according to the gradient shown in Table 1. The column was subsequently returned to its original mobile phase over the next 5 min and fluxed under these conditions for 5 additional minutes prior to the injection (20 μl) of a new sample. The absorbance was monitored at 280 nm. All the samples were analyzed in triplicate.

**Table 1:** HPLC gradient of the mobile phases used for polyphenols determination

Minutes	% A (4% acetic acid)	% B (100% methanol)
1–4	100	0
4–10	100	0
10–22	50	50
22–24	10	90
24–26	50	50
26–27	90	10

### Cell culture

The human acute monocytic leukaemia THP-1 (ATCC® TIB-202™) cell line was purchased from the American Type Culture Collection (ATCC, Manassas, USA). Cells were cultured in RPMI-1640 medium (Gibco™, Catalog No. 11875093) supplemented with 10% (v/v) heat-inactivated fetal bovine serum (FBS), 1% Penicillin–Streptomycin, 1% L-glutamine (200 mM) and 0.05 mM 2-mercaptoethanol. Cells were maintained at 37 °C and 5% CO_2_ in a humid atmosphere. All experiments were performed with cells found in an exponential growth phase.

### Cytotoxicity assay

The cytotoxic effect of *Rhus tripartita* extracts (total plant extract, rich in polyphenols, and saponin fraction extract) on THP-1 cell culture was evaluated using the colorimetric MTT assay for cell viability [25, 26]. In brief, a suspension of THP-1 cells was maintained in RPMI-1640 medium using a 96-well plate (2.0 × 105 cells/well) and incubated in humidified atmosphere at 37 °C and 5% of CO_2_ for 24 h, after which aliquots of 100 μl of medium containing different concentrations of each plant extract were added to them and incubated at 37 °C and 5% of CO_2_ for further 24 h. Subsequently, aliquots of 30 μl of MTT dye reagent (CAS 298–93-1 - Calbiochem/catalogue number 475989 Sigma-Aldrich), at the final concentration of 0.5 mg/ml, were added to each well, maintaining the 96-well plate at 37 °C and 5% of CO_2_ for further 24 h. Therefore, cells were incubated with *Rhus tripartita* extracts (total plant extract and/or saponin fraction extract) for 48 h of treatment. Sodium dodecyl sulphate (SDS; 10% v/v) was then added to each well (70 μl), followed by overnight incubation at 37 °C. This reagent was used to solubilize the MTT-formazan formed into the living cells, allowing to detect the absorbance of each formazan solution at 570 nm using a microplate reader Bio Tek® ELX800 (BioTek Instruments, Inc., Winooski, VT, USA). The percentage of cell viability for each tested extract, obtained by a comparison with untreated cell control (100% of cell viability), was used to calculate the half maximal inhibitory concentration (IC_50_), used for the successive cell treatment.

### Cell treatment with plant extracts and phenolic standards

Suspensions of THP-1 cells were incubated in 24-well plate (1.0 × 10^6^ cells/well) for 24 h in the same medium and at the same conditions previously described. Different aliquots of medium containing the relative IC_50_ concentrations of each plant extract, standard compounds and 2 μM camptothecin solution were then added to each specific well and the incubation proceeded for further 48 h at the same conditions. RNA was extracted after 48 h of in vitro culture in presence/absence of treatment. Each cell suspension was then harvested and centrifuged at 130 x g, at 26 °C for 5 min, using a Hettich Universal 32R centrifuge (Hettich GmbH & Co., Tuttlingen). The medium was then discarded and the cell pellet was washed with phosphate-buffered saline (PBS) 1X and stored in PBS 1X at − 80 °C. Each cell treatment with plant extracts and/or standard compounds was tested in triplicate. List of the THP-1 cell treatments used for all successive assays, compared to untreated control THP-1 cells (CTRL-), is as follows: *Rhus* total plant extract (RTE); saponin fraction extract (SE); rutin (R); kaempferol (K); mixture of catechin, epicatechin, and epicatechin-gallate (CEEG); ellagic acid (EA). Standard compounds (R, K, CEEG and EA) were tested at two different concentrations, based on IC_50_ values obtained from toxicity results and on IC_50_ values reported in literature for the same compounds [27–30].

### Annexin V-FITC/propidium iodide flow cytometric analysis

Analysis of phosphatidylserine externalization in apoptotic cells was determined using an Annexin V-FITC Apoptosis Detection kit (cat no. ab14085; Abcam, Cambridge, UK) according to the manufacturer’s protocol. Briefly, THP-1 cells were seeded in 6-well plates at a density of 1.0 × 10^6^ cell/well, and incubated with the different IC_50_ *Rhus tripartita* extracts at 37 °C or camptothecin 2 μM as positive control CTRL(+) at 5% CO_2_ in a humid atmosphere for 48 h. At the end of the incubation, the cells were harvested, washed twice with PBS (1X) and stained with 5 μl of FITC Annexin V (Abcam, Cambridge, UK) and 5 μl of propidium iodide (PI; Abcam, Cambridge, UK) in the dark for 5 min at room temperature. Following this procedure, cell apoptosis was analyzed on BD FACS Lyric™ flow cytometer (Becton, Dickinson and Company, Franklin Lakes, New Jersey, USA) (Ex = 488 nm; Em = 530 nm) using FITC signal detector (FL1) and PI staining by the phycoerythrin emission signal detector (FL2). Data were analyzed with the BD FACSuite™ v1.2.1 software package (BD FACSuite™, Becton, Dickinson and Company, Franklin Lakes, New Jersey, USA).

### RNA extraction, cDNA synthesis and qRT-PCR reaction

RNA isolation was carried out using TRIzol (Fisher Molecular Biology, Trevose, U.S.A.) according to manufacturer’s instructions. RNA was extracted from three biological replicates consisting of a pool of treated/non-treated cells (approximately 10^6^ cells per sample). To remove DNA, RNA samples were treated with DNase I, RNase-free (1 U μL-1) (ThermoFisher Scientific, Milan, Italy), according to manufacturer’s suggestions. cDNAs were obtained using the RevertAid First Strand cDNA Synthesis Kit (ThermoFisher Scientific) according to the manufacturer’s suggestions. Quantitative real-time polymerase chain reaction (qRT-PCR) was performed using the Maxima SYBR Green qPCR Master Mix (2X) (ThermoFisher Scientific) according to supplier’s indications, in a Rotor-Gene 6000 PCR apparatus (Corbett Robotics Pty Ltd., Brisbane, Queensland Australia). Amplification conditions were as follows: denaturation at 95 °C for 10 min, 45 cycles of 95 °C for 15 s and 60 °C for 60 s. Oligonucleotide primers, listed in Table S1, were designed using the Primer3Plus (http://www.bioinformatics.nl/primer3plus) software and further verified with OligoAnalyzer (https://eu.idtdna.com/calc/analyzer). Relative quantification was carried out using the ACT and GAPDH as reference genes [31]. The raw, background-subtracted fluorescence data provided by the Rotor-Gene 6000 Series Software 1.7 (Corbett Robotics) was used to estimate PCR efficiency (E) and threshold cycle number (Ct) for each transcript quantification and the Pfaffl method [32] was used for relative quantification of transcript accumulation. Data is represented as fold-change (FC) to untreated control in a heatmap build using the WebMeV (Multiple Experiment Viewer) tool available at http://mev.tm4.org/.

### Immunoblot analysis

Whole cell extracts were prepared by lysing cells in Laemmli buffer solution (254 mM Tris-HCl pH 6.8, 8% SDS, 0.02% bromophenol blue, 40% glycerol, 3% β-mercaptoethanol) followed by sonication. Samples were quantified and for each analysis 30 μg were boiled and loaded on a 4–20% Mini-Protean TGX Gels (Bio-Rad) for gel electrophoresis. Samples were transferred onto a nitrocellulose membrane by using the Trans-Blot Turbo transfer system (Bio-Rad). Membranes were blocked in TBS/0.1% tween/5% BSA. Immunoblotting was performed using antibodies specific for AKT (Cell Signaling, CS4685; 1:1000), AKT-pS473 (Cell Signaling, CS4060; 1:1000), S6 (Cell Signaling, CS2217; 1:1000), S6-pS240,244 (Cell Signaling, CS5364; 1:1000), γ-tubulin (Sigma Aldrich, T-6557; 1:1000) and the secondary antibodies anti-mouse-HRP (Jackson Immunoresearch, 715–005-150, 1:10000) and anti-rabbit-HRP (Jackson Immunoresearch, 711–055-152, 1:10000). Chemiluminescent signals were detected using the ChemiDoc XRS system (Bio-Rad) and quantified using the ImageJ software [33].

## Results

### Rhus tripartita contains compounds with antitumorigenic properties

The HPLC analysis on the plant extract revealed a relevant amount of polyphenols. The identified compounds showing the highest concentrations, together with the percentage of extracted saponins, are reported in Table 2.

**Table 2:** Main polyphenols identified by HPLC

polyphenols	mg/100 g DW
Catechin	2.46
Epicatechin	2.46
Epicatechin gallate	1.67
Rutin	5.01
Ellagic acid	1.20
Kaempferol	3.64
**saponins**	1.5%

The amount is expressed as mg/100 of dry weight (DW) of plant material. The amount of saponins, expressed in percentage and obtained by gravimetric analysis, is also reported

In particular, rutin is present at the highest concentration (5.1 mg/100 g DW), compared to the average of the other compounds (around 3.0 mg/100 g DW). It was observed that rutin is an active ingredient of some plant extracts used against certain cancer types [34–36]. Moreover, rutin modulates intracellular signaling cascades as evidenced by in vitro and in vivo research [37]. Catechins, epicatechin and epicatechin-gallate reached the total amount of almost 7 mg/100 g DW and were tested together, as a pool, for the remaining analyses. Catechins are potent antioxidant phytochemicals contributing to apoptosis in many cancer cell lines and were shown to target the AKT and NF-κB signaling pathways [38]. Kaempferol, found in the plant extract in a relevant amount (3,64 mg/100 g DW), has been demonstrated to compete with ATP in binding to PI3K in certain epidermal cells [39] and, generally, by neutralizing PI3K kaempferol inhibits subsequent downstream activity of AKT and its transcription factor targets. One of the most interesting observation related to the biological activity of this flavonol is that cytotoxic effects seem to target cancer cells specifically, which represents a highly desirable property [40]. Another compound identified from the obtained chromatogram was ellagic acid, at concentration of 1.20 mg/100 g DW. This compound is found to be involved in the prevention of several type of cancer [41]. The saponin content was found to be 1.5%, consistently higher compared, for example, to the amount detected in other medicinal plants from Tunisia [22]. Saponins, in general, and triterpenoid and glycosides saponins specifically, are associated with therapeutic use against numerous types of cancer, acting by inhibition of the PI3K/AKT pathway [42].

### Cytotoxic effect of Rhus tripartita extract

**Fig. 1:**
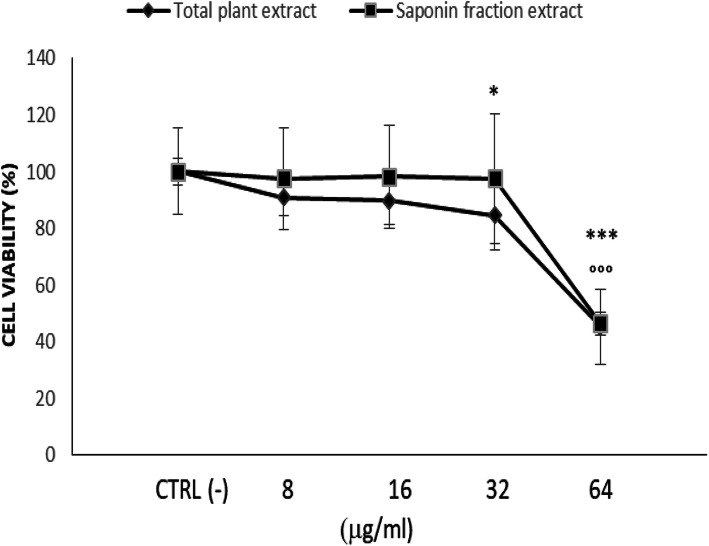
Cell viability after plant extracts treatments. *Rhus tripartita* extracts, total plant extract and saponin fraction extract, inhibited cell viability of human THP-1 cells treated with different concentrations (0, 8, 16, 32 and 64 μg/ml) of the extracts for 48 h. Data are presented as the mean ± standard deviation. (*), *p* < 0.05 total plant extract values vs. CTRL(−); (°), *p* < 0.05 saponin fraction extract values vs. CTRL(−). CTRL(−): untreated cell-control group.

The results of the MTT assay, obtained by the treatments of human THP-1 cells incubated with different concentrations (8, 16, 32, 64 μg/ml) of total plant extract and saponin fraction extract for 48 h, showed that *Rhus tripartita* extracts inhibited cell viability, revealing a cytotoxic effect, as reported in Fig. 1. The total plant extract reduced cell viability at 32 and 64 μg/ml: related to untreated cell-control group CTRL(−), *p* value was 0.051 and 6 × 10^− 5^ respectively for the two tested concentrations. The saponin fraction extract reduced cell viability at 64 μg/ml: related to the cell-control group CTRL(−), p value was 8.2 × 10^− 6^. The obtained result for IC_50_ values was 63 μg/ml for both extracts, as reported in Table 2, where the IC_50_ values (μM) of some compounds contained in the total plant extract, such as rutin, kaempferol, ellagic acid and the mixture of catechin, epicatechin and epicatechin-gallate, were also reported. Based on these results, the cell treatments for all successive biological assays were performed using total plant extract (RTE), saponin fraction extract (SE), along with the compounds rutin (R), kaempherol (K), ellagic acid (EA) and a mixture of catechin, epicatechin and epicatechin-gallate (CEEG) at the obtained IC_50_ values (Table 3).

**Table 3:** IC_50_ values

	RTE (Rhus Total Extract)	SE (Saponin Extract)	R (rutin)	K (kaempferol)	CEEG (catechin + epicatechin + epicatechin-gallate)	EA (ellagic acid)
**IC**_**50**_	**μg/ml**		**μM**			
	63.12 ± 3.24	62.85 ± 5.7	25.35 ± 1.47	29.43 ± 2.89	27.66 ± 1.26	100.34 ± 3.15
**IC**_**50**_	–	–	125	40	100	100
			[27]	[28]	[29]	[30]

The table reports IC_50_ values (μg/ml) obtained by the incubation of the THP-1 cells with different concentrations of *Rhus tripartita* total extract (RTE) and saponin fraction extract (SE). The IC_50_ values regarding the tested flavonoids (rutin, kaempferol, the mixture of catechin, epicatechin and epicatechin-gallate) contained in the total plant extract, were also reported as amount in μM and compared with the data presented in literature. The values are the average of five replicates

### Rhus tripartita extracts induce apoptosis in THP-1 cells

**Fig. 2:**
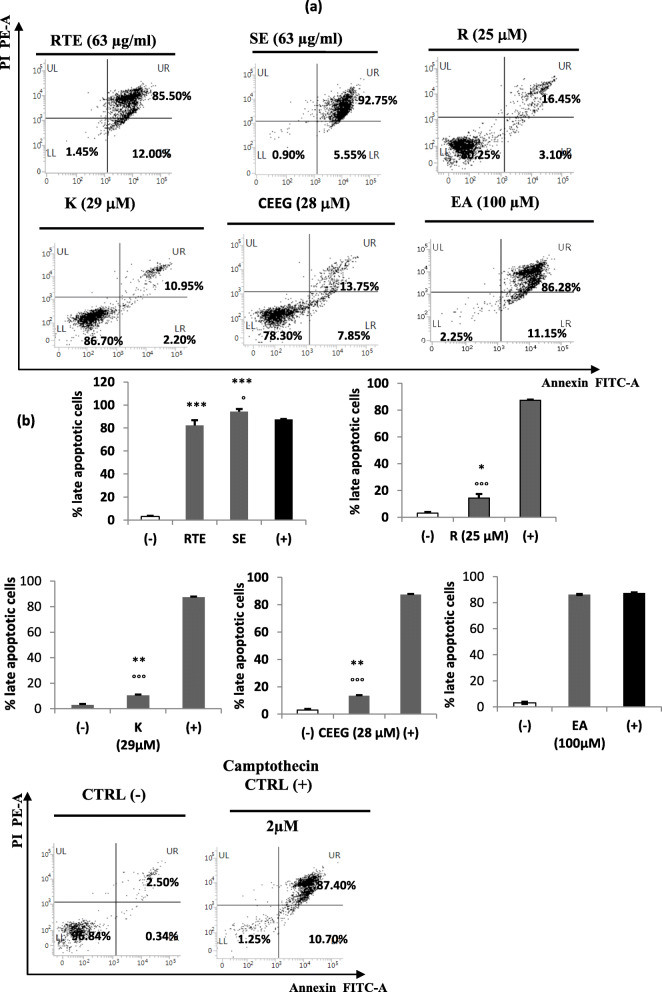
**a** Bivariate Annexin V and propidium iodide (PI) staining of THP-1cells treated with total plant extract (RTE) (63 μg/ml), saponin fraction extract (SE) (63 μg/ml), rutin (R) (25 and 125 μM), kaempferol (K) (29 and 40 μM), mixture of catechin, epicatechin, and epicatechin gallate (CEEG) (28 and 100 μM) for 48 h. The untreated cells, CTRL(−) and cells treated with camptothecin 2 μM as CTRL(+) are reported. The x-axis showed Annexin V-FITC-A (area) binding and the y-axis staining of PI labeled population (phycoerytrin PE emission signal detector). The lower left (LL) quadrant contains the viable (Annexin V- /PI-) population; the lower right (LR) quadrant contains the damaged (Annexin V+ /PI-) cells, early apoptotic cells; the upper right (UR) quadrant contains the late apoptotic cells (Annexin V+ /PI+); the upper left quadrant (UL) contains the necrotic cells and debris (Annexin V- /PI+). **b** Late apoptotic cell proportion (%). Data were presented as the mean ± standard deviation. Statistical analysis was performed using the unpaired Student’s t-test and data compared with the CTRL(−) cells (*: p < 0.05; **: *p* < 0.01; ***: *p* < 0.001) and CTRL(+) cells (°: p < 0.05; °°: p < 0.01; °°°: p < 0.001)

Annexin V/PI double staining was used to assess the proportion of apoptotic cells. The results indicated that both total plant extract (RTE) and saponin fraction extract (SE), tested at the respective IC_50_ concentration (63 μg/ml), induced late apoptosis in the THP-1 cells after 48 h (Fig. 2a), showing proportion of apoptotic cells of 82.35 ± 4.45% and 94.28 ± 2.16%, respectively. These mean values were statistically significant compared to the untreated control cells CTRL(−) (3.05 ± 0.78%) (*p*-value 0.0016 and 0.0003, respectively) (Fig. 2b). Moreover, regarding the proportion of apoptotic cells for total plant extract treatment, there was no statistically significant difference (*p*-value ≥0.05) with mean value (87.08 ± 0.46%) obtained from cells treated with the camptothecin (2 μM) (CTRL(+), an alkaloid with known antineoplastic and antitumor activity [43] (Fig. 2b). Regarding the saponin fraction extract treatment, the proportion of apoptotic cells was ever higher than that CTRL(+) (*p*-value 0.044) (Fig. 2b).

Regarding the tested compounds present in the total plant extract, the results showed that all treatments induced apoptosis (Fig. 2a). Particularly, a concentration-dependent increase in the number of late apoptotic cells was found for rutin (R) at 25 μM (14.30 ± 3.04%, *p*-value 0.037), and at 125 μM (87.68 ± 0.32%, *p*-value 5 × 10^− 5^), kaempferol (K) at 29 μM (10.55 ± 0.57%, *p*-value 0.008), and 40 μM (9.6 ± 1.41%, *p*-value 0.029), catechins, epicatechin and epicatechin-gallate (CEEG) mixture at 28 μM (13.43 ± 0.46%, *p*-value 0.004), and 100 μM (96.6 ± 0.57%, *p*-value 5.3 × 10^− 5^) (Fig. 2b). All statistical analyses were carried out comparing the effect of the treatments with the CTRL(−) data. The obtained results suggest a synergistic effect caused by the different substances found in the plant extracts.

### Inhibition of PI3K/Akt/mTOR pathway may explain Rhus tripartita extracts-induced apoptosis

**Fig. 3:**
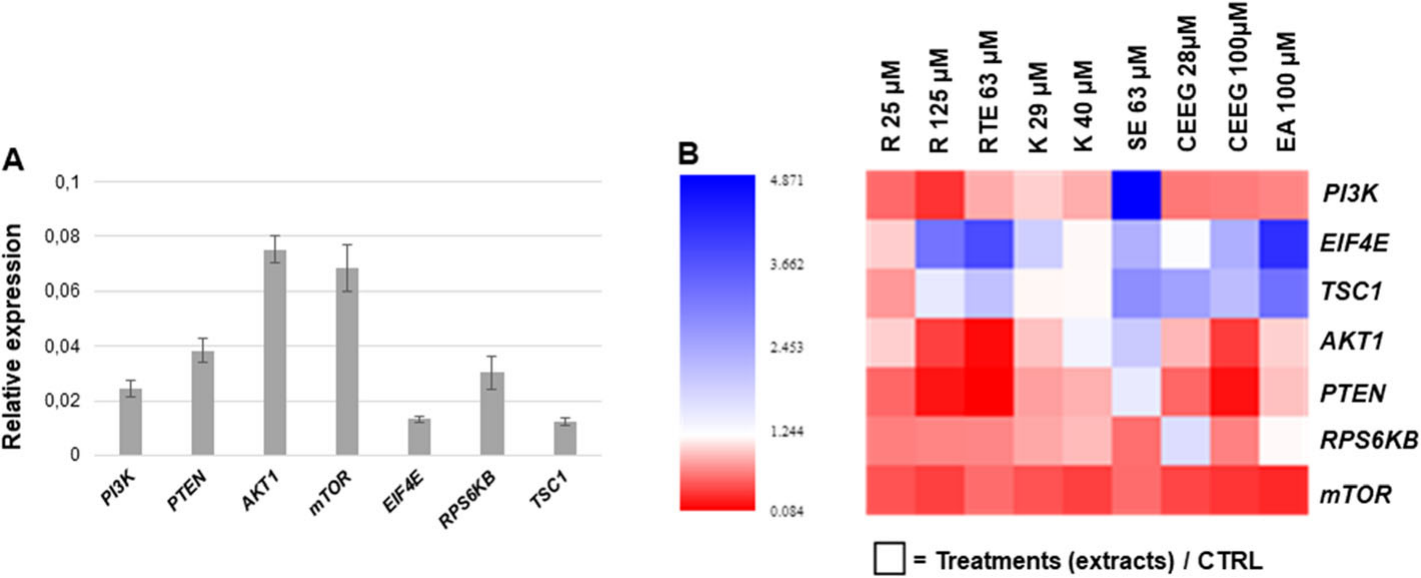
Expression profiles of genes belonging to the PI3K/AKT/mTOR pathway. **a** Relative expression of PIK3CA, PTEN, AKT1, mTOR, EIF4E, RPS6KB1, and TSC1 in CTRL (non-treated) THP-1 cells. **b** Heatmap showing changes in gene expression levels during treatments with plant extracts evidenced as fold-change (FC) to non-treated control (CTRL); PIK3CA, Phosphatidylinositol-4,5-Bisphosphate 3-Kinase Catalytic Subunit Alpha; PTEN, Phosphatase and Tensin Homolog; AKT1, AKT Serine/Threonine Kinase 1; mTOR, mechanistic Target Of Rapamycin; RPS6KB, Ribosomal Protein S6 Kinase B1; EIF4E, Eukaryotic Translation Initiation Factor 4E; TSC1, TSC Complex Subunit 1

To evaluate the effect of *Rhus tripartita* extracts on the PI3K/AKT/mTOR signaling pathways, gene expression and protein immunoblotting analyses were carried out. For gene expression analyses, a qRT-PCR approach was performed to investigate the following genes, *PI3K* (Phosphatidylinositol-4,5-bisphosphate 3-kinase), *AKT1* (AKT Serine/Threonine Kinase 1), *PTEN* (Phosphatase And Tensin Homolog), *RPS6KB* (Ribosomal Protein S6 Kinase B1), *TSC1* (TSC complex 1), *eIF4E* (Eukaryotic Translation Initiation Factor 4E) and *mTOR* (Mechanistic Target Of Rapamycin Kinase). Gene expression levels were then tested in THP-1 cells treated with *Rhus tripartita* extracts and standard phenolic compounds as described in Methods section. The expression of selected genes was represented as fold-change (FC) relative to non-treated THP-1 control (CTRL) cells. The expression profiles of the selected gene in CTRL cells are shown in Fig. 3a. It is thus possible to observe that in the absence of treatments, the most expressed genes are *AKT1* and *mTOR*. When *Rhus tripartita* extracts were applied, most of the tested genes are downregulated (Fig. 3b). For instance, *PTEN* (encoding for a tumor suppressor enzyme) resulted to be significantly downregulated by R and CEEG (at both used concentrations) as well as RTE, while the other treatments did not show significant changes (Table S2). In particular, among all the tested extracts, 25 μM R was able to significantly inhibit the expression of most tested genes. Moreover, a significative downregulation of *mTOR* gene (Fig. 3, Table S2) is evident in response to the plant extract treatments (except for K and CEEG at the highest concentrations). Similar results were obtained for *AKT1* gene (significantly downregulated by 125 μM R, RTE and CEEG treatments) and *PI3K* (significantly downregulated by most treatments except for SE where upregulation was encountered). Conversely, a significant upregulation of *eIF4E* (in response to 125 μM R, RTE, SE, 100 μM CEEG, EA treatments) and *TSC1* (in response to RTE, SE, both concentrations of CEEG and EA treatments) was observed.

Specific studies conducted in the THC-1 line indicated that downregulation of PTEN mediated by miRNA-32 resulted in enhanced expression of M2 macrophage polarization [44]. Similarly, miR-8, overexpressed in many cancer cells lines, including THC-1, suppresses PTEN expression, thus promoting apoptosis. When a specific inhibitor of miR-8 was used, the enhanced expression of PTEN (no longer downregulated by the activity of the miRNA itself), resulted in induced apoptosis [45]. The *EIF4E* gene is known to be overexpressed in many human malignancies, being a crucial player in mRNA translation, export and stability; its function is controlled by interactions with protein cofactors in concert with many signaling pathways, including PI3K, mTOR, and AKT1 [46]. Other studies have shown that its transcriptional upregulation in AML is correlated with the NF-κB dependent pathway [47]. On the other side, it is well known that TSC1 forms a complex with TSC2 to regulate cellular metabolism and homeostasis through inhibition of mTORC1. Inactivation of these genes result in elevated activity of mTORC1 which controls activation of p70 S6 kinase and eIF4E to regulate transcriptional expression and translation of critical proteins involved in metabolic processes [48]. Hence, the upregulation of *TSC1* under the imposed treatments may be well correlated with *mTOR* downregulation. Indeed, mTOR is part of two complexes, mTORC1 and mTORC2. mTORC1 activity can be measured by analyzing the phosphorylation of the direct downstream target S6K1 on Thr389 or phosphorylation of ribosomal subunit S6 [49, 50], while mTORC2 phosphorylates AKT on the primary phosphorylation site at Ser473 [51].

**Fig. 4:**
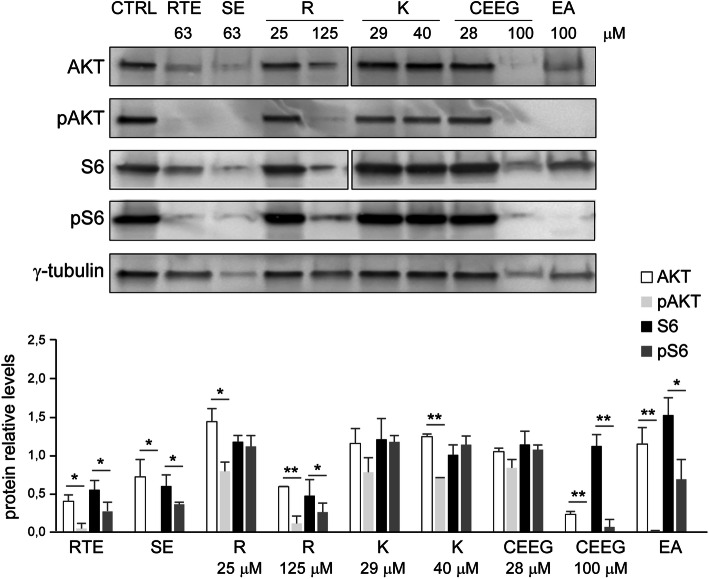
S6 and AKT activation is inhibited by Rhus tripartita extracts. **a** Immunoblot analysis with antibodies specific for AKT, phosphorylated AKT (pAKT), S6 or phosphorylated S6 (pS6) of THP-1 whole cell extracts before (CTRL) or after treatment with the indicated concentration of the *Rhus tripartita* total extract (RTE), saponin extracts (SE), rutin (R), kaempferol (K), a mixture of catechin, epicatechin and epicatechin-gallate (CEEG) or ellagic acid (EA) for 48 h. Full-length blots from the two gels used for this figure are shown in Supplementary Fig. 1 (Fig. S1). **b** The protein levels were quantified, normalized to the γ-tubulin loading control levels and expressed as fold increase relative to CTRL cells. Bars indicate SEM (*n* = 3). For each treatment, the levels of phosphorylated proteins were compared to the levels of the unphosphorylated form. Asterisks (*), indicates Student’s t-test with p < 0.05 (*), p < 0.01 (**)

To further investigate the role of mTOR pathway in *Rhus tripartita* extracts-induced apoptosis, the activation of S6 and AKT proteins was examined in THP-1 cells by immunoblot assay using antibodies directed against the phosphorylated Ser240–244 of S6 and the phosphorylated Ser473 of AKT. As shown in Fig. 4, AKT showed strong levels of phosphorylation (pAKT) in untreated THP-1 cells (CTRL) and upon treatment using the lowest concentration of R, K (both concentrations) and CEEG. Conversely, compared to CTRL cells and the corresponding unphosphorylated signal, S6 phosphorylation level resulted significantly diminished after 48 h treatment with RTE, SE, R, 40 μM K, 100 μM CEEG and EA. Concomitantly, the ribosomal subunit S6 showed a dramatic decrease of phosphorylation at Ser473 (pS6) upon cell treatment with RTE, or SE, 125 μM R, 100 μM CEEG and EA, if compared to CTRL cells and the corresponding unphosphorylated signal. Altogether, these results indicate that *Rhus tripartita* extracts can attenuate the phosphorylation of mTORC1 substrate S6Ser2040–244 and mTORC2 downstream target AKT-Ser473 in acute monocytic leukaemia THP-1 cells, suggesting that the cell cycle arrest and apoptosis induced by *Rhus tripartita* extracts involve the PI3K/AKT/mTOR signaling pathway.

## Discussion

Recently, a large number of literature reports on the potential use of plant extracts to inhibit cancer cell proliferation by targeting components of the PI3K/AKT/mTOR signaling pathway [52–56] indicating how this is an important field of research in continuous expansion. The PI3K/AKT/mTOR signaling pathway is one of the major intracellular pathways, tightly regulated under normal physiological conditions, and it is recognized to regulate both normal and malignant hematopoiesis [11]. Hyperactivation of the PI3K-AKT signaling axis drives uncontrolled cell proliferation and survival in several types of cancer while its inhibition is considered the most important factor in determining the response to chemotherapy and the outcome of AML [57]. It has been reported that patients with AML and hyperactivated AKT signaling in AML cells exhibit a worse prognosis and shorter survival time compared with patients with normal levels of activated AKT [57, 58]. Drugs targeting this pathway may prove useful in the treatment of different types of malignancy. Moreover, AML treatment remains a major challenge due to poor efficacy of the current chemotherapeutics [59]. Therefore, development of novel and more effective treatment strategies is urgently needed. While progress has been made in the understanding of the genetic and molecular biology of the disease, the standard of care for patients had only changed minimally over the past 40 years. Recently, rapid movement of potentially useful agents from bench to bedside has translated either into new therapies recently approved or in clinical trials. These therapies include improved chemotherapies, mutationally targeted inhibitors, pro-apoptotic agents, microenvironment targeting molecules, cell cycle checkpoint inhibitors, and epigenetic regulators is an aggressive, heterogeneous, myeloid malignancy [60]. Moreover, the main therapeutic challenge in the treatment of myeloid leukaemia is the development of strategies that maximize the induction of leukaemia cell apoptosis before resistance to chemotherapy sets in. According to this aspect, the targeted therapy with new drugs may have an important role for disease treatment. Although several targeted therapies have recently been approved for AML, research is always looking for new drugs that can be used in AML treatment (for example inhibitors or regulators of gene expression) (In: https://www.cancer.gov/types/leukemia/research). Herbal remedies have been widely used in traditional medicine to support the treatment of different types of cancer. Moreover, the use of plant-derived products in the treatment of cancer may contribute to reduce adverse and toxic side effects, often observed using specific synthetic drugs. For this reason, many plants showing very promising anticancer activities in vitro and in vivo are studied, but most of the times their active anticancer principle have yet to be evaluated. Therefore, investigating plants as potential resources for antitumor agents is an increasingly important topic in cancer research. This underlines the growing interest related to the use of plant extracts as support therapeutics in this regard. For instance, flavonoids and isoflavones extracted from *Tephroseris kirilowii* and *Astragalus membranaceus*, well-known herbs used in Chinese traditional medicine, resulted in induced apoptosis by inhibiting the PI3K/AKT/mTOR pathway in various types of human breast cancer cells [52, 53]. More recently, rosemary (*Rosmarinus officinalis*) total extracts resulted in inhibited phosphorylation/activation of AKT and mTOR, being indicated as having potent anticancer properties [55].

In this study, the apoptotic effect of a total extract and some compounds found at relevant concentrations in *Rhus tripartita*, was tested in a THP-1 cell cultures. *Rhus tripartita* is a local pre-Saharan Tunisian plant that often grows in non-agricultural regions and it is widely used in food industry and in modern and traditional medicine. Antimalarial, antiviral, antimicrobial, antitumorigenic and atherosclerosis properties have been reported for this species [61]. Few articles report on the phytochemical composition of this plant [62] but there are still no studies that investigate which compounds are responsible for the biological activity or possible mechanisms involved. On the other side, it is complicated to compare the biochemical profiles of the *Rhus tripartita* described in literature, due to the different maturity stage and the climatic and environmental conditions that often influence the compositional quality and the quantity of several metabolites of the plant. In this work, the main phytochemicals present in *Rhus tripartita* extracts were determined and quantified. In particular, relevant concentrations of rutin and catechins were found; also, the percentage of saponins resulted to be significantly high. These compounds, and in general many of the flavonoids present in the total extract, play an important role in inhibition of the PI3K/AKT/mTOR signaling pathways. In instance, rutin, present in several aromatic and medicinal plants, is an active ingredient and has significant activity against breast, hepatic and colon cancer [35, 37]. Among the observed activities, rutin is also able to exert inhibitory effect on AKT and induce apoptosis in cancer cells. Likewise, catechins have been reported to induce apoptosis in many cancer cell lines, in some of which downregulating PI3K and AKT [63]. Data shown in the present article confirm this evidence. All treatments induced apoptosis and the number of late apoptotic cells was found to increase for rutin, kaempferol and mix of catechins according to the used concentrations. Similarly, total plant extract (RTE) and saponin fraction extract (SE) induced late apoptosis in the THP-1 cells after 48 h, suggesting a synergistic effect of the different compounds found in the plant extract. To investigate if *Rhus tripartita* induction of apoptosis affects the expression of genes related to the PI3K/AKT/mTOR signaling pathway (*PI3K, AKT1, PTEN, RPS6KB, TSC1, eIF4E*, and *mTOR*), a qRT-PCR analysis was carried in treated/untreated THP-1 cells. Analysis of the *PI3K*, *AKT1* and *mTOR* apoptosis-linked genes demonstrated that treatment with rutin (R), at the highest concentration, and CEEG mixture, were able to significantly downregulate the expression of these genes whereas *eIF4E* and *TSC1* genes were upregulated in THP-1 cells treated with these extracts. Except for saponins and kaempferol at the highest concentration, all the treatments strongly decrease the expression level of *AKT1*, especially the total plant extract (RTE) and the highest concentrations of rutin and catechins mix. This agrees with other studies showing downregulation of PI3K/AKT/mTOR in response to treatments with plant phytochemicals extracted from *Ricinus communis* [64], *Alpinia nantoensis* [65], *Taraxacum officinale* [66]. Moreover, the *eIF4E* and *TSC1* genes, acting as negative regulators of mTOR [8], were upregulated in our study, thus strengthening once more the inhibitory effect that *Rhus tripartita* extracts have on THP-1 cell proliferation. In fact, accordingly with the observed results regarding the *mTOR* downregulation, almost all the treatments, especially EA, CEEG mix and SE, sharply increase the expression level of *TSC1*. To further explore the effect of *Rhus tripartita* extracts and active compounds on the mTOR signaling pathway, the expression of AKT protein, S6 and their phosphorylated forms was investigated. It was observed that, with exception of SE, which decrease the phosphorylated form of the protein, western blotting analysis confirms that the highest concentrations of the CEEG mix and R, together with EA and RTE, inhibit the AKT phosphorylation. Total plant extract seems to decrease the expression of p-AKT and p-S6, indicating that plant extract may exert anticancer effects partly by inhibition of the PI3K/AKT/mTOR signaling pathway. Herein, to the best of our knowledge, it was demonstrated for the first time that *Rhus tripartita* total extract can inhibit cell proliferation and induce apoptosis in THP-1 cells by altered expression of *PI3K, AKT, mTOR* and *PTEN* and inhibition of AKT and S6 phosphorylation. These results suggest that the compounds contained in the total plant extracts, more than the single compound, are effective to suppressing the proliferation of AML THP-1 cells and that this may be partially mediated by the downregulation of the PI3K/AKT/mTOR signaling pathway. In conclusion, *Rhus tripartita* extracts represents useful candidates for development of supporting anticancer therapies, specifically in the treatment of AML, due to its ability to kill the cells and then positively regulate apoptotic mechanism.

## Supplementary Information

**Additional file 1.**

**Additional file 2.**

## Data Availability

The data supporting the findings of this study are available from the corresponding author of this manuscript upon reasonable request and with permission of all the authors.
