# Supplementary material for: The polyphenol/saponin-rich Rhus tripartita extract has an apoptotic effect on THP-1 cells through the PI3K/AKT/mTOR signaling pathway
Source: BMC Complement Med Ther. 2021 May 27;21:153. doi: 10.1186/s12906-021-03328-9 (PMC8161611; doi:10.1186/s12906-021-03328-9)
Supplement: Supplementary file 1 — Additional file 1. [file 12906_2021_3328_MOESM1_ESM.docx]

Title: The polyphenol/saponin-rich Rhus tripartita extract has an apoptotic effect on THP-1 cells through the PI3K/AKT/mTOR signaling pathway

Authors: Hajer Tlili ^1^, Anca Macovei ^2^, Manuela Lanzafame ^3^, Daniela Buonocore ^2^, Anita Lombardi ^3^, Hanen Najjaa ^1^, Maurizia Dossena ^2^, Andrea Pagano ^2^, Manuela Verri ^2^, Abdelkarim Ben Arfa ^1^, Mohamed Neffati ^1^ and Enrico Doria ^2 *^

Affiliations

^1^ Laboratory of Pastoral Ecosystems and Valorization of Spontaneous Plants and Microorganisms, Institute of Arid Regions (IRA), Medenine, Tunisia - [hajer1992tlili@gmail.com](mailto:hajer1992tlili@gmail.com),

^2^ Department of Biology and Biotechnology “L. Spallanzani,” University of Pavia, Pavia, Italy; [anca.macovei@unipv.it](mailto:anca.macovei@unipv.it)

^3^ Institute of Molecular Genetics IGM CNR, Pavia, Italy; manuela.lanzafame@gmail.com

***** Correspondence: enrico.doria@unipv.it; Tel.: +39 0382986388

**Table S1.**  Oligonucleotide sequences used for qRT-PCR analysis.

| **Gene** | **Forward primer (5’-3’)** | **Reverse primer (5’-3’)** |
| --- | --- | --- |
| *PI3K* | GGTTGTCTGTCAATCGGTGACTGT | GAACTGCAGTGCACCTTTCAAGC |
| *PTEN* | GGTTGCCACAAAGTGCCTCGTTTA | CAGGTAGAAGGCAACTCTGCCAAA |
| *AKT1* | TTCTGCAGCTATGCGCAATGTG | TGGCCAGCATACCATAGTGAGGTT |
| *mTOR* | GCTTGATTTGGTTCCCAGGACAGT | GTGCTGAGTTTGCTGTACCCATGT |
| *EIF4E* | GAACGAACCCTTCCTTCCGAATGA | AGGGCGAAGGTGGCTTTTATTTCC |
| *RPS6KB1* | ACTGTAGTGTTGACTGCCTGACCA | TAGCCAGCCAATCACAGTGCTCAT |
| *TSC1* | GCAGCGTGACACTATGGTAACCAA | AGTTCTATCCGCAGCTCCGCAAT |
| *GAPDH* | TGCACCACCAACTGCTTAGC | GGCATGGACTGTGGTCATGAG |
| *ACT* | GACAGGATGCAGAAGGAGATTACT | TGATCCACATCTGCTGGAAGGT |

**Table S2**. Gene expression data normalized to reference gene (*GAPDH, ACT*) as revealed by the qRT-PCR analysis. The statistical significance calculated as per Students t-test, comparing the non-treated control (CTRL) with each treatment (R 30μM, R 125μM, RTE, K 29μM, K 40μM, SE, CEEG 28μM, CEEG 100μM, EA) is shown with asterisk, where *p <0.05, **p <0.01, ***, p <0.001.

|  | **CTRL** | **R 30μM** | **R 125μM** | **RTE** | **K 29μM** | **K 40μM** | **SE** | **CEEG 28μM** | **CEEG 100μM** | **EA** |
| --- | --- | --- | --- | --- | --- | --- | --- | --- | --- | --- |
| ***PI3K*** | 0.024 ± 0.003 | 0.012 ±0.001** | 0.007 ± 0.002** | 0.019 ± 0.006 | 0.022 ± 0.005 | 0.019 ± 0.002 | 0.117 ± 0.005*** | 0.014 ± 0.0004** | 0.014 ± 0.0002** | 0.015 ± 0.001* |
| ***eIF4E*** | 0.012 ± 0.001 | 0.012 ± 0.0007 | 0.042 ± 0.007** | 0.050 ± 0.017* | 0.024 ± 0.005* | 0.014 ± 0.003 | 0.030 ± 0.006* | 0.015 ±  0.001 | 0.030 ± 0.0009** | 0.055 ± 0.006*** |
| ***TSC1*** | 0.012 ± 0.001 | 0.008 ± 0.0002* | 0.018 ± 0.004 | 0.025 ± 0.004 | 0.013 ± 0.002 | 0.013 ± 0.001 | 0.034 ± 0.001*** | 0.031 ± 0.001*** | 0.026 ± 0.004** | 0.039 ± 0.006** |
| ***AKT1*** | 0.075 ± 0.005 | 0.071 ±0.014 | 0.026 ± 0.010*** | 0.009 ± 0.0007*** | 0.067 ± 0.015 | 0.098 ± 0.012* | 0.145 ± 0.018*** | 0.063 ±  0.013 | 0.024 ± 0.007*** | 0.071 ± 0.026 |
| ***PTEN*** | 0.038 ± 0.004 | 0.019 ±0.005** | 0.006 ± 0.001** | 0.003 ± 0.001** | 0.028 ± 0.004 | 0.031 ± 0.002 | 0.055 ± 0.014 | 0.019 ± 0.005* | 0.005 ± 0.001** | 0.033 ± 0.007 |
| ***RPS6KB*** | 0.030 ± 0.006 | 0.018 ± 0.002* | 0.019 ± 0.001* | 0.019 ± 0.003 | 0.023 ± 0.004 | 0.025 ± 0.003* | 0.016 ± 0.001* | 0.049 ± 0.001* | 0.018 ±  0.005* | 0.0133 ± 0.002 |
| ***mTOR*** | 0.068 ± 0.018 | 0.029 ± 0.005* | 0.023 ± 0.009* | 0.036 ± 0.010* | 0.029 ± 0.005* | 0.023 ± 0.009 | 0.036 ± 0.010** | 0.025 ± 0.005* | 0.020 ±  0.003 | 0.017 ± 0.0007** |
