# Supplementary material for: The polyphenol/saponin-rich Rhus tripartita extract has an apoptotic effect on THP-1 cells through the PI3K/AKT/mTOR signaling pathway
Source: BMC Complement Med Ther. 2021 May 27;21:153. doi: 10.1186/s12906-021-03328-9 (PMC8161611; doi:10.1186/s12906-021-03328-9)
Supplement: Supplementary file 2 — Additional file 2. [file 12906_2021_3328_MOESM2_ESM.pdf]

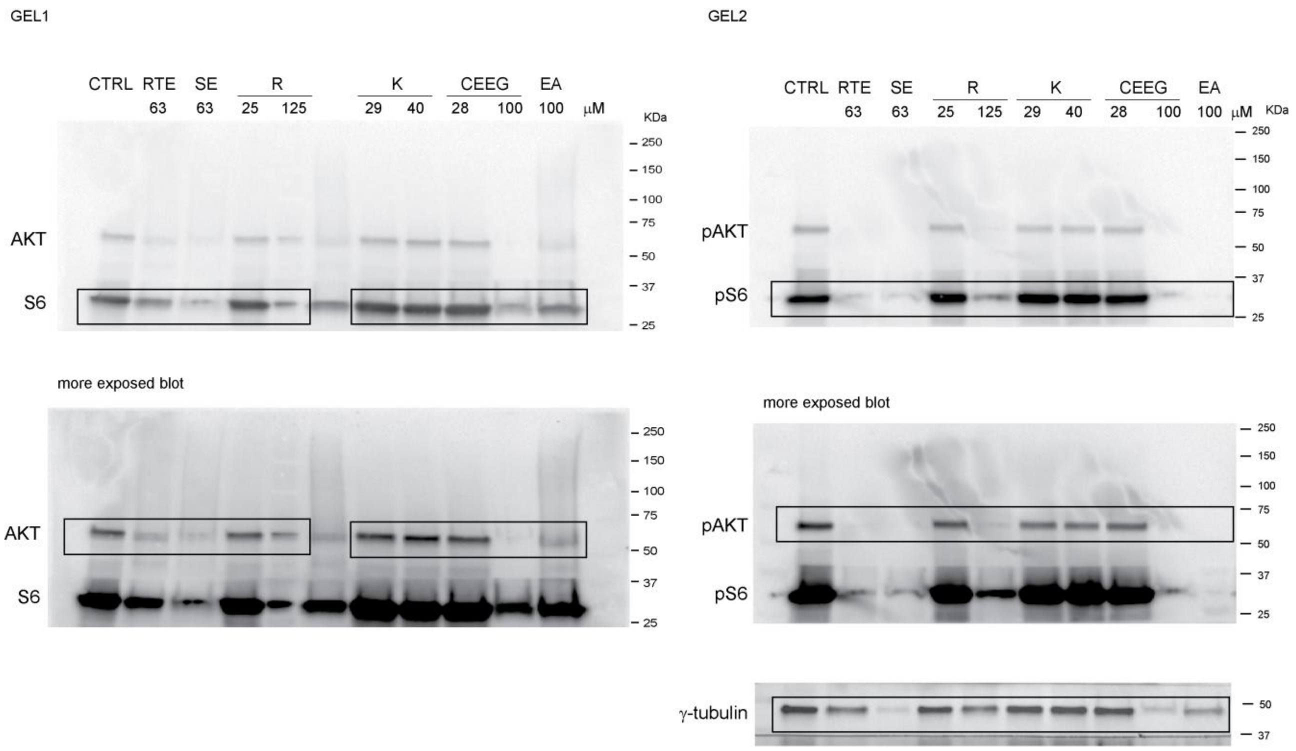

Supplementary Figure 1. Full-length blots from the two gels used for figure 4. Gel 1 was used to develop AKT and S6, Gel 2 was used to develop the phosphtylated forms of AKT and S6 (pAKT and pS6 respectively). Boxes indicate the cropped areas used in figure 4. Different exposures are indicated.
